# Genetic Characterization of Infectious Bursal Disease Viruses Associated with Gumboro Outbreaks in Commercial Broilers from Asyut Province, Egypt

**DOI:** 10.1155/2014/916412

**Published:** 2014-02-09

**Authors:** Moemen A. Mohamed, Kamal E. S. Elzanaty, Bakhit M. Bakhit, Marwa M. Safwat

**Affiliations:** Department of Poultry Diseases, Faculty of Veterinary Medicine, Asyut University, Asyut 71515, Egypt

## Abstract

Ten infectious bursal disease virus (IBDV) field strains were isolated from 15 broiler flocks located in various parts of Asyut, Egypt. Seven strains were subjected to comparative sequencing and phylogenetic analyses to help provide optimal control program for protection against IBDV infection. Sequence analysis of a 530 bp hypervariable region in the VP2 gene revealed that the rate of identity and homology was around 95.6~99.1%. Sequence characterization revealed the 7 strains identified as vvIBDV with the four amino acids residues typical of vvIBDV (242I, 256I, 294I, 299S). The BURSA-VAC vaccine was the nearest vaccine in sequence similarity to the local examined IBDV strains followed by CEVACIBDL then Bursine plus and Nobilis Gumboro indicating its probable success in the face of incoming outbreaks when using these vaccines. Phylogenetic analysis revealed that the presence of three clusters for the examined strains and are grouped with reference very virulent IBDVs of European and Asian origin (Japanese and Hong Kong) strains suggesting the different ancestors of our isolates. The antigenic index showed a number of changes on the major and minor hydrophilic antigenic peaks of the virus surface structures indicating a new genetic evolution of the surface structure epitopes that may lead to vaccination failure and reemergence of the disease.

## 1. Introduction

Infectious bursal disease (IBD) is an acute, highly contagious viral disease of young birds characterized mainly by severe lesions in the bursa of Fabricius causing fatal condition and immunosuppression in chickens [1]. Infectious bursal disease virus (IBDV) belongs to the family Birnaviridae and has nonenveloped capsid. Since the first report in 1989, IBDV has two subtypes; the first one is variant and the other is the classical subtype that has been subdivided into 3 pathotypes: attenuated, virulent, and very virulent (vvIBDV) [2]. These vvIBDV strains were reported to break through high levels of maternal antibodies in commercial flocks, causing up to 60–100% mortality rates in chickens and producing lesion typical of IBDV [3].

The IBDV genome is divided into segments A and B: segments A (3.4 kb) and B (2.8 kb). The large segment A encodes 4 viral proteins, the two capsid proteins VP2 (48 kDa) and VP3 (32–35 kDa), the viral protease VP4 (24 kDa), and a nonstructural protein VP5 (17–21 kDa), while the smaller segment B encodes VP1 (90 kDa), an RNA-dependent RNA polymerase. Expression/deletion studies have shown VP2 aa positions 206 to 350 to represent a major conformational, neutralizing antigenic domain called hyper variable region (HVR) [4], which includes the most variable region important for cell antigenic and pathogenic variation. Most exchanges of amino acid residues in VP2 occur in the four hydrophilic loops of the viral capsid [5]. These exchanges indicate that selective pressure for the evolution of IBDV is directly focused on the capsid regions that are immediately exposed to the immune system [6].

In spite of the most commercial broiler chicken flocks that are vaccinated against IBD, severe outbreaks were reported in Egypt, caused high mortalities, and have become a priority problem [7–9]. To expand our understanding of the molecular epidemiology of IBDV in Asyut, Egypt, the coding sequences of HVR of VP2 of 7 isolates were analyzed and compared with other viral isolates from other countries to better understand the evolution of IBDV and to design appropriate immunisation regimes.

## 2. Materials and Methods

### 2.1. Sample Collection

A total of 15 edematous and hemorrhagic bursal tissues were collected from different broiler chicken flocks of 23 and 27 days of age exhibiting the clinical and pathological features of IBD from the south of Asyut Province. The broilers were vaccinated at their 11th and 22th days with a live attenuated IBD vaccine that used an intermediate standard strain. The specimens were kept frozen at −80°C until processing.

### 2.2. Virus Identification

#### 2.2.1. Viral RNA Extraction

The collected bursal tissues were homogenized with phosphate-buffered saline with penicillin and streptomycin (1000 ug/mL each) as a 30% homogenate and the suspension was vortexed prior to three rounds of freeze thawing.

The total viral RNA in the samples was extracted from 250 uL of homogenized bursa tissue using 750 uL of TRIZOL LS reagent (Invitrogen), according to the manufacturer's protocol. The total RNA was quantified by spectrophotometry (OD) and stored at −80°C.

#### 2.2.2. Reverse Transcription Procedure

RT-PCR was done using QIAGEN One-Step RT-PCR by amplification of the hypervariable region of VP2 with the following forward and reverse primers: the sense primer VP2F (5′-CGC CAG GGT TTT CCC AGT CAC GAC AAC AGC CAA CAT CAA CG-3′) and the antisense primer VP2R (5′-TCA CAC AGG AAA CAG CTA TGA CGC TCG AAG TTR CTC ACC C-3′). The expected fragment size was 723 bp [11]. 10 uL of a reaction buffer, 2.0 uL of dNTP mix, 2 uL of enzyme mixture, 100 pmol of each oligonucleotide, and 5 uL of RNA were mixed with RNase-free water to a final volume of 50 uL. All ingredients were kept on ice during handling. The RT-PCR program is as follows: 30 min at 50°C (RT reaction); 94°C for 2 min (initial PCR activation); 39 three-step cycles of 94°C for 30 sec, 58°C for 1 min, and 68°C for 2 min; and 68°C for 7 min (final extension).

#### 2.2.3. IBDV Pathotype Identification Using Restriction Endonuclease Digestion (RE) [12]

For the differentiation of cvIBDV strains from vvIBDV, 10 uL of the resulting 723 bp RT/PCR products was digested with fast digest* SacI* restriction enzyme according to the manufacturer's instructions (Thermo Scientific, MA, USA), allowing 30 minutes for digestion to ensure that all reactions went to completion. The digested PCR products were visualized on 1.5% agarose gels stained with ethidium bromide. The sizes of the RFLP bands were estimated by comparing them with a 100 bp plus DNA ladder included on the gel (Invitrogen, USA).

### 2.3. Sequencing and Phylogenetic Analyses of the HVR of VP2

To define the genpotype of the 7 chosen isolates, the amplified 723 bp RT-PCR products of VP2 gene for each chosen strain were purified by QIAquick PCR (Qiagen Inc., Valencia, CA, USA) following manufacturer's instructions. The purified RT-PCR products were sequenced by the dideoxy-mediated chain-termination method using ABI prism 377-Perkin Elmer automated sequencer at Lab Technology Company in Cairo, Egypt.

The VP2 hypervariable region that located between nucleotide 730 and 1260 bp sequences [10] of the seven chosen field isolates was determined, then submitted to GenBank, and deposited in the GenBank database under the following accession numbers: (strain-1 (KF316959); strain-2 (KF316960); strain-3 (KF316961); strain-4 (KF316962); strain-5 (KF316963); starin-6 (KF316964); strain-7 (KF316965)).

To establish a better framework to discuss the molecular epidemiology of the chosen IBDV isolates, the nucleotide and amino acid sequences of the 7 chosen strains were analysed in conjunction with sequence data from other strains obtained from GenBank (http://www.ncbi.nlm.nih.gov/Genbank). MegAlign program of Lasergene 7.0 (DNASTAR Inc., WI, USA) was used for analyses, nucleotide sequence editing, amino acid predictions, and sequence alignments of the VP2-HVR.

## 3. Results

### 3.1. Virus Identification Using RT-PCR

**Figure 1:**
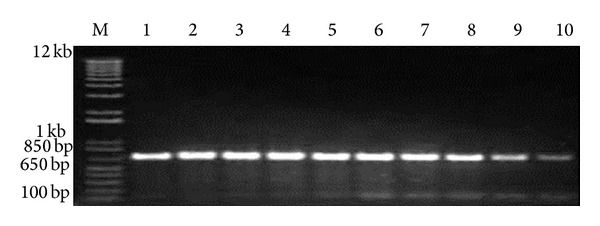
RT-PCR amplified products of 723 bp fragment of VP2 gene of Assiut field isolates from lanes 1–8 of IBDV in 1% agarose gel electrophoresis. M: molecular marker (1 KB plus DNA ladder).

RT-PCR analysis revealed that 10 out of 15 examined samples were positive by RT-PCR which produced a band of 723 bp corresponding to the partial amplification of VP2 gene of IBDV (Figure 1).

### 3.2. IBDV Pathotype Identification by RE Digestion

**Figure 2:**
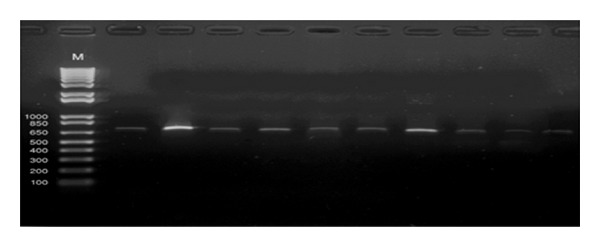
Restriction fragment length polymorphism (RFLP) profile of the examined isolates of VP2 gene of IBDV after digestion with restriction enzyme *SacI* in 1% agarose gel.

The previously obtained RT/PCR products of size 723 bp were digested with restriction enzyme *SacI* and the results revealed that all the examined strains assigned to be very virulent IBDVs that are indicated by the missing restriction site of the *SacI* in the examined strains (Figure 2).

### 3.3. Nucleotide and Amino Acid Deduced Sequence Analyses

*Nucleotide Sequence Identity.* Results revealed that none of the chosen examined strains were 100% similar and the divergence rates were ranged from 0.9% (between strains 7 and 2) to 4.3% (between strains 3 and 6) (Table 1).

**Figure 3:**
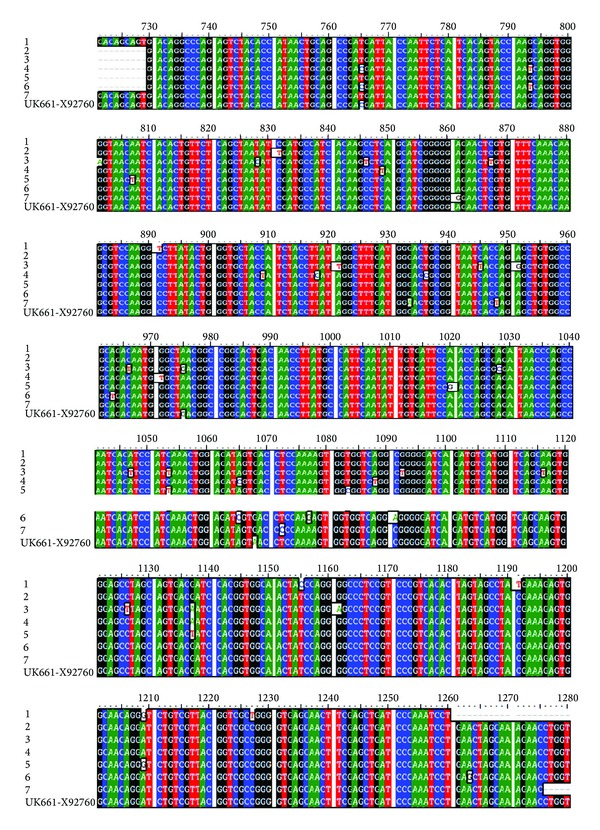
Nucleotide alignment of the VP2 hypervariable region of the seven examined IBDV isolates with the reference vvIBDV (UK661). Continued color in the columns indicates position where the sequence is identical to that of the hypervirulent UK661 strain. The unshaded cells show the nucleotidic differences among examined viruses. The vvIBDV signature nucleotides are indicated by arrows and bold, underlined letters in the UK661 sequence (C767, G795, T827, T830, C833, C857, A866, A897, T905, T908, T989, A1011, G1094, and A1115).

Figure 3 shows that all the examined isolates had genetic characteristics consistent with the reference very virulent strains and were more closely related to the vvIBDV reference isolate UK661. Twelve nucleotide positions (779G, 827T or A, 830T, 833C, 857C, 866A, 897A, 905T, 908T, 989T, 1011A, and 1094G) previously reported to be conserved in the reference UK-661 are found in our strains that indicated its very virulence genotype.

The observed identity percentages of the examined strains with reference vvIBDV were 97–99.3% with the Uk661, 96.6–99.1% with OKYM and 96.7–99% with the vvIBDV HK46 (Table 1). Also the chosen examined strains had high similarity percentages (94.8% to 99.1%) with the early Egyptian isolates (Table 2).

Table 3 shows the divergence among examined isolates and some of the commercially available vaccines and revealed that the most nearest vaccine in similarity was BURSA-VAC with divergence rate 4.9% in contrast to 9.3% was noted with Nobilis Gumboro 228E vaccine.

**Figure 4:**
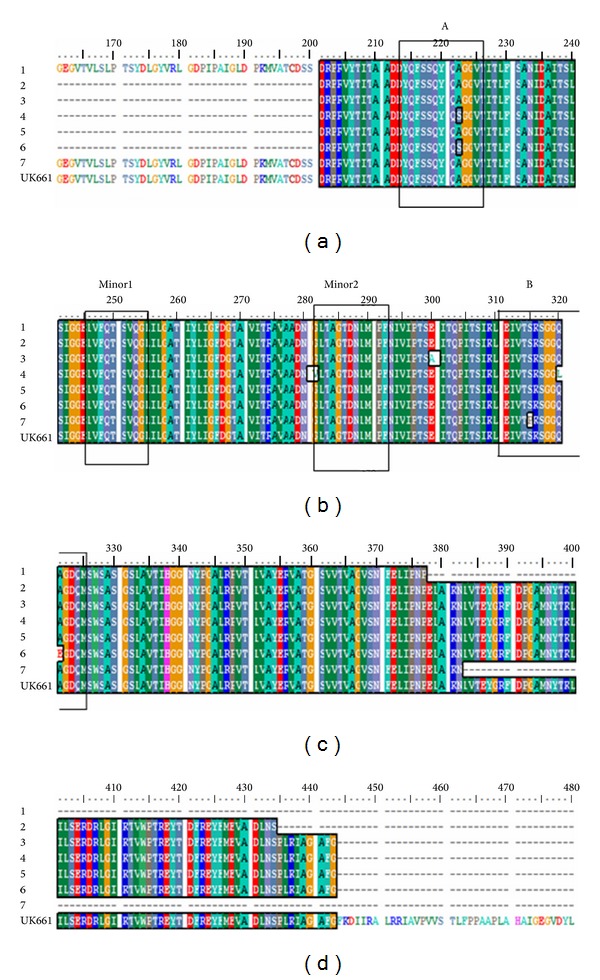
Alignment of deduced amino acid sequence of the VP2 variable domain from aa positions 170 to 480 (numbering according to Bayliss et al., 1990, in seven field strains: 1, 2, 3, 4, 5, 6, and 7 compared with reference IBDV strain (UK661) [10]. The hydrophilic peaks (regions critical for antigenicity) are boxed.

*Amino Acids Sequence Analysis.* To determine if trends exist in IBDV genome evolution, analysis of the deduced amino acid substitutions in the variable VP2 (vVP2) region of chosen strains was performed (Figure 4). The analyzed region included 159 amino acid residues, from position 201 to 377. It was found that none of the local examined isolates are of vaccinal or attenuated origin due to absence of 253-Histidine and 284-Threonine mutations that are typically found in attenuated vaccine strains [2]. Figure 4 revealed that all of the examined strains showed the characteristic of vvIBDV amino acid substitutions at residues 222A, 242I, 256I, 294I, and 299S except strains 4 and 6 has serine at (222S) in substitution of alanine (222A). Also all of the examined isolates have the serine-rich heptapeptide SWSASGS that was found next to the second hydrophilic region 326–332 that confirmed the nature of highly virulence among the chosen strains.

Figure 4 shows amino acid (aa) substitution mutations in the major and minor hydrophilic peaks. The first substitution was observed at position 222 in virus strains 4 and 6 in the major hydrophilic peak region A, the second and third one at the position 281 and 320 in the minor hydrophilic peak region 2 and major one region B, respectively in strain 4. In addition to fourth one in strain 6 at position 321 in the major peak region B.

**Figure 5:**
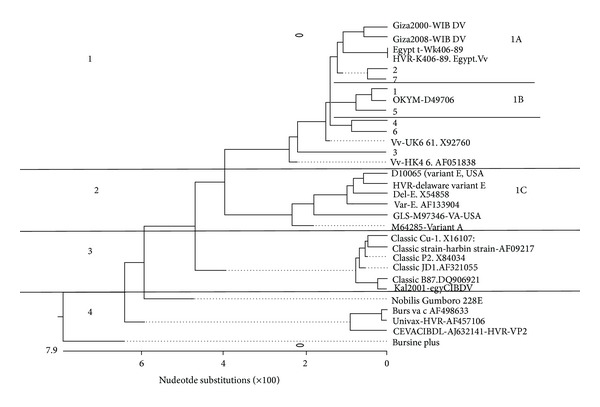
Phylogenetic consensus tree of the studied IBDV strains. The nucleotide (nt) sequences encoding the VP2 variable domain in seven examined field strains (1, 2, 3, 4, 5, 6, and 7) were compared with several very virulent, classical, variant, or cell culture adapted viruses from various geographical origins.

*Phylogenetic Analysis of Nucleotide Sequences.* As shown in Figure 5 the examined isolates were grouped in the same cluster of the vv strains group; then they divided into three subclusters tentatively called 1A that contains strains 2 and 7, 1B that contains strains 1 and 5, and 1C cluster that contains strains 3 and 4 which indicates that our strains have three different ancestors (European, Japanese, and Hong Kong).

**Figure 6:**
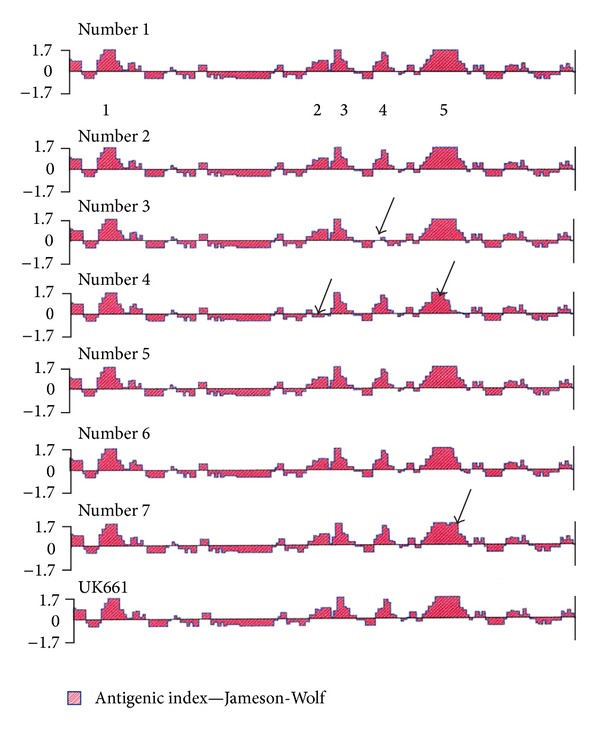
Peaks identified as potential antigenic sites within the deducted amino acid sequence of the hypervariable region of VP2 gene from amino acid residues 183 to 356 (numbering system of Bayliss et al., 1990 [10]). The seven examined strains are being compared. The numbers below strain are as follows: number 1 indicates the five major antigenic peaks described. The arrow shows the missing antigenic peaks numbers 4 and 2 in strains 3 and 4, respectively, as well as changes in the topography of major antigenic peak number 5 in the strains 4 and 7.

*Antigenic Index*. The potential antigenic sites within the predicted aa sequence of the region of HVR of VP2 from positions 183 to 356 were analyzed in the seven local isolates in comparison with UK661 reference strain (Figure 6). Similar profiles of antigenic peaks were noted for strains 1, 2, 5, and 6 as seen with the European UK661. However, strains 3 and 4 exhibited a different profile in which a missed one peak no. 4 and 2 were observed respectively. Also the topography of the major antigenic peak number 5 in the strains 4 and 7 had been changed.

## 4. Discussion

One of the major problems observed in the field is the frequent outbreaks of IBD in spite of the extensive use of available IBD vaccines. This failure in vaccination could be due to changes in VP2 (which is major protective antigen of IBDV) that may result from immunological pressure or genetic reassortment between more than one strain [13].

In this study we undertook the first molecular characterisation of infectious bursal disease virus in Asyut Province. The VP2 of IBDV was targeted to diagnose these chosen strains to track evolutionary changes at the molecular level by sequencing.

Fifteen bursal samples were collected from different farms with clinical signs and lesions that were characteristic of Gumboro infection and analyzed for presence and determination of the genotype of IBDv. Out of 15 examined samples, 10 samples were identified as positive for IBDV using the partial amplification of VP2 by RT-PCR (Figure 1). Then, the pathotype of the 10 IBDV strains was determined using *SacI*-PCR-RFLP and the results showed that all recovered IBDVs are very virulent pathotype due to absence of the *SacI* site in those strains (Figure 2), as shown by Zierenberg et al., 2001 [12].

Molecular evaluation of the hypervariable region of the 7 chosen isolates was conducted to compare their nucleotide and amino acid sequences with those of other countries and other parts of the world. A comparative alignment and phylogenetic analysis of the hypervariable domain of the VP2 region helped to group the IBDV isolates into different pathogenic subgroups. Results obtained from molecular analysis of the 7 chosen IBDV isolates demonstrated that these strains have similarities with previously characterized Egyptian isolates in terms of molecular features (Table 2 and Figure 5).

Twelve out of the 14 nucleotide positions (Figure 3) and four amino acid residues (Figure 4), that is, characteristics to very virulent pathotype (Figures 3 and 4), found in our examined strains [2, 4, 14] indicated that these isolates have a very virulent pathotype. Results of phylogenetic analysis of the VP2 gene based on the nucleotide sequences (Figure 5) demonstrated that the chosen 7 examined viruses have high similarity to vvIBDV detected in other parts of the world and have different origins.

As shown in Figure 4 several substitutions mutation were observed, at position 222 in the major hydrophilic peak region A within virus strains 4 and 6, at the position 281 and 320 in the minor hydrophilic peak region 2 and major hydrophilic region B respectively in strain 4 and at position 321 in the major hydrophilic peak region B. Isolate 3 exhibited the amino acid residue 300 Ala (Figure 4) other than the rest of the examined strains that exhibited glutamic acid at this position.

Isolate 3 exhibited the amino acid residue 300 Ala (Figure 4) other than the rest of the examined strains that exhibited glutamic acid at this position. After an extensive search in the NCBI protein database, this amino acid residue appeared only in other geographically distant vvIBDV isolates, such as very virulent isolates from Bangladesh [17], Nigeria [11], India, and Nepal [18]. Glutamic acid is characterized by negatively charged (acidic) R group, whereas the R group in alanine is nonpolar (hydrophobic). It is probable that this change from negative charge to nonpolar may modify the protein folding or the interaction with other molecules that may change the topography of the neutralizing epitopes that may lead to vaccination failure [19].

Antigenic index that reflects the influence of several different parameters such as hydrophilicity, surface probability, backbone flexibility, and secondary structure [20, 21] was determined for the 7 sequenced strains. As shown in Figure 6, there are missing antigenic peaks numbers 4 and 2 in strains 3 and 4, respectively, as well as changes in the topography of major antigenic peak number 5 in the strains 4 and 7.

In summary, this study demonstrated that the circulating genotype in the sites of study of Asyut Province is very virulent strains. The 7 chosen vvIBDVs cluster phylogenetically with vvIBDVs from European, Japan and Hong Kong. vVP2 sequences are responsible for the determination of antigenicity and pathogenicity of the virus. The mutations in vVP2 were noticed in our isolates especially in the minor and major hydrophilic peaks. Amino acid changes in this region could affect these characteristics of vvIBDV strains and the control of the disease in the future.

## Figures and Tables

**Table 1:** Nucleotide similarity (%) of the HVRs of VP2 of the examined strains with reference vvIBDV strains (OKYM (Japan), HK46 (Hong Kong), and UK661 (European).

Percent identity												
	**1**	**2**	**3**	**4**	**5**	**6**	**7**	**8**	**9**	**10**		
Divergence												
**1**	∗	98.9	95.9	97.2	98.1	97.8	98.0	99.3	98.6	98.7	**1**	1
**2**	1.1	∗	96.7	98.2	98.6	98.4	99.1	99.1	99.0	99.3	**2**	2
**3**	4.3	3.4	∗	95.9	96.2	95.7	96.2	96.6	96.7	97.0	**3**	3
**4**	2.9	1.9	4.2	∗	97.6	98.3	97.4	97.9	97.8	98.4	**4**	4
**5**	2.0	1.4	3.9	2.4	∗	97.7	97.9	98.9	98.2	98.6	**5**	5
**6**	2.2	1.6	4.4	1.7	2.3	∗	97.9	98.1	98.0	98.6	**6**	6
**7**	2.0	0.9	4.0	2.6	2.1	2.2	∗	98.5	98.5	98.5	**7**	7
**8**	0.7	0.9	3.5	2.1	1.1	1.9	1.5	∗	99.0	98.9	**8**	OKYM-D49706
**9**	1.5	1.0	3.4	2.2	1.8	2.0	1.5	1.0	∗	98.9	**9**	Vv-HK46, AF051838
**10**	1.3	0.7	3.1	1.7	1.4	1.4	1.5	1.1	1.1	∗	**10**	Vv-UK661, X92760
	**1**	**2**	**3**	**4**	**5**	**6**	**7**	**8**	**9**	**10**		

*NB, it is mean the percent of identity is 100 or zero percent divergence between isolates.

**Table 2:** Nucleotide similarity (%) of the HVRs of VP2 of the examined strains with 3 Egyptian IBDV strains.

Percent identity													
	**1**	**2**	**3**	**4**	**5**	**6**	**7**	**8**	**9**	**10**	**11**		
Divergence													
**1**	∗	98.9	95.9	97.2	98.1	97.8	98.0	98.8	97.6	96.6	98.7	**1**	1
**2**	1.1	∗	96.7	98.2	98.6	98.4	99.1	99.6	98.1	97.2	99.3	**2**	2
**3**	4.3	3.4	∗	95.9	96.2	95.7	96.2	96.4	95.2	94.8	97.0	**3**	3
**4**	2.9	1.9	4.2	∗	97.6	98.3	97.4	97.8	96.7	95.8	98.4	**4**	4
**5**	2.0	1.4	3.9	2.4	∗	97.7	97.9	98.4	96.9	96.1	98.6	**5**	5
**6**	2.2	1.6	4.4	1.7	2.3	∗	97.9	98.5	96.9	96.0	98.6	**6**	6
**7**	2.0	0.9	4.0	2.6	2.1	2.2	∗	98.4	97.6	96.8	98.5	**7**	7
**8**	1.3	0.4	3.7	2.2	1.6	1.5	1.6	∗	98.3	97.5	99.2	**8**	Egypt-Vvk406-89
**9**	2.5	1.9	5.0	3.4	3.2	3.2	2.5	1.7	∗	98.9	98.1	**9**	Giza2000-VvIBDV
**10**	3.5	2.9	5.4	4.3	4.0	4.1	3.3	2.5	1.1	∗	97.3	**10**	Giza2008-VvIBDV
**11**	1.3	0.7	3.1	1.7	1.4	1.4	1.5	0.8	1.9	2.7	∗	**11**	Vv-UK661, X92760
	**1**	**2**	**3**	**4**	**5**	**6**	**7**	**8**	**9**	**10**	**11**		

*NB, it is mean the percent of identity is 100 or zero percent divergence between isolates.

**Table 3:** Nucleotide similarity (%) of the HVRs of VP2 of the examined strains with 4 vaccinal IBDV strains.

Percent identity													
	**1**	**2**	**3**	**4**	**5**	**6**	**7**	**8**	**9**	**10**	**11**		
Divergence													
**1**	∗	98.9	97.2	95.9	98.1	97.8	98.0	92.5	94.5	94.4	94.0	**1**	1
**2**	1.1	∗	98.2	96.7	98.6	98.4	99.1	93.4	95.3	94.8	94.4	**2**	2
**3**	2.9	1.9	∗	95.9	97.6	98.3	97.4	92.1	93.4	92.6	92.6	**3**	3
**4**	4.3	3.4	4.2	∗	96.2	95.7	96.2	91.4	93.3	92.4	91.4	**4**	4
**5**	2.0	1.4	2.4	3.9	∗	97.7	97.9	91.7	93.2	92.7	92.5	**5**	5
**6**	2.2	1.6	1.7	4.4	2.3	∗	97.9	92.1	93.7	93.1	93.3	**6**	6
**7**	2.0	0.9	2.6	4.0	2.1	2.2	∗	92.4	94.1	94.0	93.8	**7**	7
**8**	8.0	7.0	8.5	9.3	9.0	8.5	8.2	∗	95.8	95.5	94.2	**8**	Bursine plus
**9**	5.8	4.9	7.0	7.2	7.2	6.7	6.2	4.4	∗	98.2	96.3	**9**	Bursvac-AF498633
**10**	6.0	5.5	7.9	8.2	7.8	7.4	6.4	4.7	1.8	∗	96.0	**10**	CEVACIBDL-AJ632141-HVR-VP2
**11**	6.4	5.8	7.9	9.2	8.0	7.1	6.6	6.1	3.8	4.1	∗	**11**	Nobilis Gumboro 228E
	**1**	**2**	**3**	**4**	**5**	**6**	**7**	**8**	**9**	**10**	**11**		

*NB, it is mean the percent of identity is 100 or zero percent divergence between isolates.
